# Patterns of multimorbidity in working Australians

**DOI:** 10.1186/1478-7954-9-15

**Published:** 2011-06-02

**Authors:** Libby Holden, Paul A Scuffham, Michael F Hilton, Alexander Muspratt, Shu-Kay Ng, Harvey A Whiteford

**Affiliations:** 1 School of Medicine, Griffith University; University Drive Meadowbrook, Queensland 4131, Australia; 2 Queensland Centre for Mental Health research, Queensland Health; Level 3 Dawson house, The Park, Wacol, Queensland 4076, Australia; 3 University of Queensland, School of Population Health; Herston Road Herston, Queensland 4006, Australia

## Abstract

**Background:**

Multimorbidity is becoming more prevalent. Previously-used methods of assessing multimorbidity relied on counting the number of health conditions, often in relation to an index condition (comorbidity), or grouping conditions based on body or organ systems. Recent refinements in statistical approaches have resulted in improved methods to capture patterns of multimorbidity, allowing for the identification of nonrandomly occurring clusters of multimorbid health conditions. This paper aims to identify nonrandom clusters of multimorbidity.

**Methods:**

The Australian Work Outcomes Research Cost-benefit (WORC) study cross-sectional screening dataset (approximately 78,000 working Australians) was used to explore patterns of multimorbidity. Exploratory factor analysis was used to identify nonrandomly occurring clusters of multimorbid health conditions.

**Results:**

Six clinically-meaningful groups of multimorbid health conditions were identified. These were: factor 1: arthritis, osteoporosis, other chronic pain, bladder problems, and irritable bowel; factor 2: asthma, chronic obstructive pulmonary disease, and allergies; factor 3: back/neck pain, migraine, other chronic pain, and arthritis; factor 4: high blood pressure, high cholesterol, obesity, diabetes, and fatigue; factor 5: cardiovascular disease, diabetes, fatigue, high blood pressure, high cholesterol, and arthritis; and factor 6: irritable bowel, ulcer, heartburn, and other chronic pain. These clusters do not fall neatly into organ or body systems, and some conditions appear in more than one cluster.

**Conclusions:**

Considerably more research is needed with large population-based datasets and a comprehensive set of reliable health diagnoses to better understand the complex nature and composition of multimorbid health conditions.

## Background

The term 'comorbidity' was first used in 1970 by Feinstein (as cited by Kessler et al, 2001 [1]) and by van den Akker et al [2,3] to refer to situations where an individual has two or more physical and/or mental health conditions. More recently, the term multimorbidity was introduced [2-4]. Although comorbidity and multimorbidity are both used to describe two or more health conditions, a distinction is made between these two terms. Comorbidity is used when an index condition of interest is being discussed, and multimorbidity is used when no reference condition is considered [4]. Although these distinctions often are not clearly applied, and both terms are used interchangeably in the literature, we will use this definition of these terms in this paper. Sometimes health conditions can be comorbid purely by chance; however, certain comorbidity clusters can also occur at higher than chance levels[1].

International and Australian research demonstrates the prevalence of comorbidity or multimorbidity as increasing significantly with age [3-6], indicating that patients with multimorbidity in general practice represent the rule, rather than the exception[5,7,8]. For example, an Australian study exploring data obtained through 305 general practitioners in 2005 reported that the prevalence of multimorbidity increased with age, with 83% of surveyed patients aged 75 years or older having multimorbidity [6].

The study of patterns of multimorbidity is a new field. While there is a growing body of evidence regarding the prevalence of comorbidity and multimorbidity [3-5,9], most studies use either a count of the number of comorbidities, such as the Charlson Index [10], or a Cumulative Illness Rating Scale (CIRS), which groups conditions by body systems affected [6,11-13]. These methods do not use statistical approaches to identify the nonrandom cluster patterns of individual health conditions into groups of multimorbid conditions, perhaps due to the limitations of statistical methods to date. Most statistical packages that can perform exploratory factor analysis (EFA) require the data to be in a continuous format, but health conditions are usually dichotomously represented; that is, the person either has the condition or does not.

The objective of this study was to use software and statistical analysis methods that allow for the dichotomous nature of disease data to identify nonrandomly occurring clusters of multimorbid health conditions. Identifying clusters of multimorbidity is important due to rising health care costs associated with servicing an increasingly aging population with complex health care needs. Health service providers need to better understand the complexity of the health status of consumers to ensure more strategic and tailored health care is provided.

## Methods

### Data

The Australian Work Outcomes Research Cost-benefit (WORC) project (http://www.qcmhr.uq.edu.au/worc/) provides a large cross-sectional data set of 78,430 working Australians to explore clusters of nonrandomly occurring multimorbid health conditions.

Study sample: Employees of 58 large Australian-based companies were invited to participate in the WORC study. The survey was undertaken between October 2004 and December 2005.

Study measures: The Health and Productivity Questionnaire (HPQ) from the World Health Organization [14] was used to collect self-reported health status on 22 health conditions. The Kessler 6 (K6) [15], a validated measure of psychological distress, which is included within the HPQ, was used to collect psychological distress data. In total, 23 conditions were explored for multimorbidity patterns in this study. The following health conditions were included in the analyses, as these were available in the HPQ: arthritis, asthma, back/neck pain, cancers (excluding skin cancer), skin cancers, chronic obstructive pulmonary disease (COPD) (including chronic bronchitis and emphysema), cardiovascular disease (CVD), psychological distress (defined as a K6 score of 13 and above [16]), drug and alcohol problems, diabetes, fatigue (including sleep problems), high blood pressure, high cholesterol, injury (workplace injury requiring medical treatment), migraine (and severe headache), obesity (using self reported height and weight to calculate body mass index), bladder problems, heartburn, irritable bowel disorder, ulcers, osteoporosis, or other chronic pain. Self-reported health status was coded for this study as "yes" if respondents reported having the condition and were either currently or had previously received professional treatment for that condition, and "no" if they reported never having the condition. Respondents were excluded if they reported having a condition but never received treatment, as these respondents may have incorrectly self-diagnosed the health problem. An average of 0.05% respondents were excluded for each condition.

### Statistical analysis

Exploratory factor analysis was performed in the software package *Mplus *[17], which accommodates for dichotomous variables by calculating tetrachoric correlations among the variables. When working with tetrachoric correlations, there are no assumptions concerning the shapes of the frequency distributions, and as a consequence, there is no need to be concerned that some distributions are skewed. Factor solutions for the one-factor solution through to the eight-factor solution were explored. The optimal number of factors was determined after applying a number of rules and indices: the scree test (in a plot of eigenvalues against factor number, a kink in the plot gives the optimal number of factors [18]); the eigenvalues-greater-than-one rule [18]; standardized root mean square residual (SRMR), which should be less than 0.05 [19]; comparative fit index (CFI) and Tucker Lewis index (TLI), both of which should be greater than 0.95 [19]; and a rule which says that more than two items should contribute to the definition of a factor [17]. An orthogonal quartimin rotation was applied to facilitate interpretation of factor loadings.

## Results

The sample demographic characteristics are listed in Table 1. The sample included part-time, full-time, and casual workers. In the sample, 65% were female and 35% male. The two largest age groups were those aged 30-44 years and those aged 45-59 years, comprising 80% of the sample. Those aged less than 18 years and over 70 years were excluded from the study, as these age groups are not usually in the Australian workforce (0.2% deleted). A total of 71% was married or cohabiting, 69% had no children, 48% had completed a tertiary qualification, and 53% earned $50,000 or more per year.

**Table 1:** Sample Demographic Characteristics

Demographic Independent Variable	N	%
AGE ^¥^	78410	

18-29 years		17

30-44 years		43

45-59 years		37

60-70 years		3

SEX	78430	

Female		65

Male		35

MARITAL STATUS	78212	

Separated, divorced, widowed, never married		29

Married or cohabitating		71

NUMBER OF CHILDREN	78209	

Nil		69

1-3 children		28

4 or more children		3

EDUCATION LEVEL	78430	

Did not complete high school		14

Completed high school		10

Some college		27

Completed college or university		48

ANNUAL WAGE β	76778	

≤$29,999 pa		13

$30,000-39,999 pa		14

$40,000-49,999 pa		21

$50,000-74,999 pa		36

$75,000-99,999 pa		10

≥ $100,000 pa		7

¥: only persons aged 18-70 included in analysis; β: excludes hourly rate < $7.50 ph in case fortnightly income reported instead of annual income

**Figure 1:**
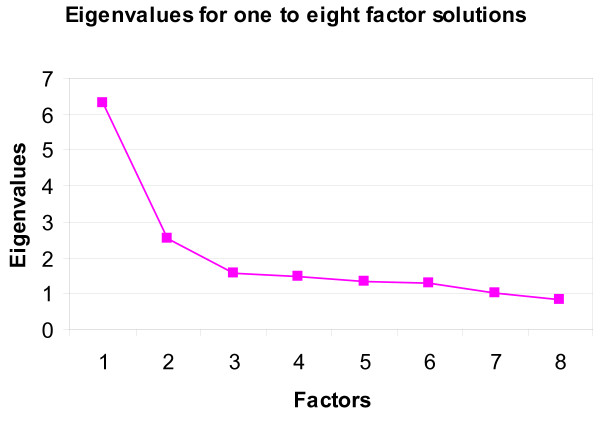
**Scree Test with Eigenvalues for Range of Solutions**.

We obtained all solutions from the one-factor solution to the eight-factor solution. The scree test (Figure 1) suggests that the optimal number of factors is two or three. However, all of the other indices suggest a larger number of factors. The CFI and TLI goodness-of-fit statistics (Table 2) suggest a five-factor solution, whereas SRMR suggests a six-factor solution. The eigenvalues-greater-than-one rule suggests a six- or perhaps a seven-factor solution. However, the seven-factor solution does not meet the requirement of having a minimum of three items in a factor, and so is not considered ideal. Therefore, the six factor solution was selected. Table 3 provides the loadings for the six-factor solution (loadings exceeding the cut-off of ± 0.40 appear in bold).

**Table 2:** Exploratory Factor Analysis Goodness-of-fit Statistics for the One Factor Solution through to the Eight Factor Solution

Factors	CFI	TLI	df	SRMR
1	0.471	0.713	94	0.128

2	0.742	0.852	164	0.091

3	0.845	0.903	150	0.079

4	0.923	0.947	136	0.062

5	0.950	0.962	122	0.051

6	0.965	0.971	114	0.043

7	0.981	0.982	100	0.032

8	0.987	0.986	86	0.027

**Table 3:** Loadings for the Six-factor Solution following an Exploratory Factor Analysis Based on a Polychoric Correlation matrix

	Factor 1	Factor 2	Factor 3	Factor 4	Factor 5	Factor 6
Arthritis	**0.578**	0.255	**-0.545**	0.346	**0.401**	0.378

Asthma	0.116	**0.950**	-0.207	0.168	0.055	0.202

Back/neck problems	0.274	0.206	**-0.747**	0.127	0.071	0.261

COPD	0.348	**0.634**	-0.248	0.276	0.261	0.275

CVD	0.276	0.067	-0.078	0.377	**0.773**	0.259

diabetes	0.088	0.125	-0.064	**0.883**	**0.567**	0.129

High cholesterol	0.204	0.132	-0.134	**0.439**	**0.807**	0.276

Fatigue	0.234	0.230	-0.298	**1.000**	**0.461**	0.245

High blood pressure	0.083	0.158	-0.177	**0.521**	**0.769**	0.259

Injuries	0.203	0.101	-0.393	0.167	0.025	0.139

Migraine	0.141	0.280	**-0.562**	0.159	-0.036	0.269

Obesity	-0.025	0.197	-0.255	**0.502**	0.368	0.216

Drug & alcohol	0.334	0.229	-0.293	0.388	0.088	0.387

Psychological distress	0.083	0.121	-0.295	0.271	-0.033	0.218

Cancer (not skin)	0.319	0.102	-0.157	0.138	0.140	0.189

Irritable bowel	**0.424**	0.302	-0.391	0.230	0.021	**0.653**

Other chronic pain	**0.587**	0.230	**-0.614**	0.361	0.166	**0.472**

Ulcer	0.252	0.224	-0.311	0.173	0.216	**0.934**

Heartburn	0.260	0.326	-0.376	0.212	0.322	**0.841**

Allergies	0.237	**0.800**	-0.377	0.127	0.053	0.379

Bladder problems	**0.469**	0.247	-0.312	0.323	0.188	0.370

Skin cancer	0.374	0.116	-0.122	0.139	0.253	0.138

Osteoporosis	**0.614**	0.232	-0.279	0.150	0.227	0.214

CFI: 0.965, TLI: 0.971; SRMR 0.043; Loadings are shown after the application of the Quartimax orthogonal rotation; Loadings are bolded if they exceed ± 0.4

The following factors were identified:

• Factor 1: arthritis, osteoporosis, other chronic pain, bladder problems, and irritable bowel

• Factor 2: asthma, COPD, and allergies

• Factor 3: back/neck pain, migraine, other chronic pain, and arthritis

• Factor 4: high blood pressure, high cholesterol, obesity, diabetes, and fatigue

• Factor 5: CVD, diabetes, fatigue, high blood pressure, high cholesterol, and arthritis

• Factor 6: irritable bowel, ulcer, heartburn, and other chronic pain

## Discussion

Some conditions appear in more than one factor. (One reason exploratory factor analysis was used for this study is that it allows for more than one factor per condition.) Previous studies that use statistical methods to explore relationships of multimorbid conditions or clusters of organ systems have also found that some conditions appear in more than one factor [6,20,21]. Of the 23 conditions available for analysis in our study, we found chronic pain to be in three of the six clusters; diabetes, high blood pressure, and high cholesterol to be in the same two of the six clusters; and arthritis and irritable bowel to be in two different clusters.

We found that health conditions do not cluster neatly into organ or body system, as has been assumed in the methods underpinning the CIRS [22]. A study by Britt et al [20] demonstrates this. They explored patterns of multimorbidity and found that groups of individuals fit into between two and eight combinations of CIRS domains [20].

Only one other study was found that explored patterns of multimorbidity among individual health conditions [21]. A study by Cornell et al included more than 1.3 million primary care patients cared for by the Veterans Health Care System with two or more comorbidities and categorized 45 health conditions. Similarities exist between our fifth group of health conditions and Cornell's "metabolic cluster," the cluster that had the highest degree of association in their study. They reported that 83% of their sample fell into this cluster; three of the conditions in this cluster were also represented in our fifth factor [21]. Differences between the study by Cornell et al and this study include statistical method (Cornell's methods of cluster analysis relies on prevalence, so conditions with low prevalence will be underrepresented), sample size and composition (Cornell's sample was much larger, and all study participants had two or more health conditions; our sample included people well enough and young enough to attend work), and the number of health conditions (these were greater in the Cornell study). These differences may account for discrepancies in the cluster composition between the two studies.

Other existing measures either calculate a comorbidity score based on the number of coexisting conditions, with some weights applied to adjust for severity of condition, such as the Charlson Index [10,23-25], or calculate the impact on functional status, such as the Functional Comorbidity Index [26]. Studies that explore multimorbidity tend to use one of these instruments to determine comorbidity and/or multimorbidity. Because the Charlson Index requires hospital admission data and accurate International Classification of Diseases 10^th ^Revision (ICD-10) records, many of these studies do not reflect the population as a whole. Our study uses those still in the workforce, perhaps skewing to those in better health in the community. Further research is required in this area to determine prevalence and structure of multimorbid clusters of health complaints occurring in Australia.

This study adds to the only other available study [21] that uses statistical methods on a group of individual health conditions to explore nonrandom clustering of multimorbidity. With an increasingly aging population and evidence that comorbidity and multimorbidity increase with age [3-5], combined with rising health care costs associated with new procedures and treatments, a better understanding of how health conditions cluster together will enable better care management of individuals with chronic and complex diseases.

There are some limitations to our study that need to be considered, and extrapolation of these findings to the general population should be done with caution. This is an opportunistic sample of willing employees from 58 large organizations. The response rate was low (22%). A comparison of respondents and nonrespondents was not possible, so the implications of the poor response rate are not known. For example, only those at work during the data collection period responded. People on extended sick leave or out of the workforce are not represented. The sample also has overrepresentation of females. The self-reported nature of health conditions, and the number and type of health conditions available also need to be considered. For example, there is an absence of some high-cost conditions, such as kidney disease. Therefore, extrapolation of these findings to the general population should be done with caution. The findings are relevant, however, to those sectors and groups where the demographic profile is similar. Fatigue, which may be either chronic or acute, was included in the model. Fatigue is mostly acute, so one might question whether it should be included. However, the results demonstrate that fatigue is included in two of the multimorbidity groupings, highlighting its importance for inclusion in multimorbidity analyses.

## Conclusions

This study identified clinically meaningful clusters of multimorbid health conditions that do not fall neatly into organ or body systems. Some conditions appear in more than one cluster. Few studies are available that use statistical methods to explore patterns of multimorbidity in a group of individual health conditions. A large population-based sample with reliable diagnosis data at an individual level is required.

## Abbreviations

CFI: Comparative fit index; CIRS: Cumulative Illness Rating Scale; COPD: Chronic obstructive pulmonary disease; CVD: Cardiovascular disease; HPQ: Health and Productivity Questionnaire (World Health Organization); K6: Kessler 6 screening tool for psychological distress; TLI: Tucker Lewis index; SRMR: Standardized root mean square residual; WORC: Work Outcomes Research Cost-benefit project (Australian-based)

## Competing interests

The authors declare that they have no competing interests.

## Authors' contributions

LH developed the methods for this study in collaboration with co-investigators, conducted data analysis with help from statistician, wrote first draft and revisions to paper, corresponding author; PAS assisted with developing methods, advised on statistical analysis methods, and reviewed drafts of paper; MH coordinated data collection and reviewed drafts of paper. AM and SKN helped with the data analysis, and HAW chief investigator of parent study which collected the data used in this study and reviewed drafts of paper. All co-authors read and approved the final manuscript.

## References

[B1] Kessler RC Comorbidity 2001 Amsterdam, NY: Elsevier Science Ltd

[B2] van den Akker M Buntinx F Metsemakers J Knottnerus J Marginal impact of psychosocial factors on multimorbidity: results of an explorative nested case-control study Social Science & Medicine 2000 50 1679 1693 10.1016/S0277-9536(99)00408-6 10795973

[B3] van den Akker M Buntinx F Metsemakers JF Roos S Knottnerus JA Multimorbidity in general practice: prevalence, incidence, and determinants of co-occurring chronic and recurrent diseases J Clin Epidemiol 1998 51 367 375 10.1016/S0895-4356(97)00306-5 9619963

[B4] van den Akker M Buntinx F Roos S Knottnerus J Problems in determining occurrence rates of multimorbidity Journal of Clinical Epidemiology 2001 54 675 679 10.1016/S0895-4356(00)00358-9 11438407

[B5] Fortin M Bravo G Hudon C Vanasse A Lapointe L Prevalence of multimorbidity among adults seen in family practice Annals of Family Medicine 2005 3 223 10.1370/afm.272 15928225 PMC1466875

[B6] Britt H Harrison C Miller G Knox S Prevalence and patterns of multimorbidity in Australia Medical Journal of Australia 2008 189 72 77 18637770 10.5694/j.1326-5377.2008.tb01919.x

[B7] Saltman D Sayer G Whicker S Co-morbidity in general practice Postgraduate Medical Journal 2005 81 474 480 10.1136/pgmj.2004.028530 15998827 PMC1743309

[B8] Fortin M Hudon C Bayliss E Soubhi H Lapointe L Caring for body and soul: the importance of recognising and managing psychological distress in persons with multimorbidity International Journal of Psychiatry in Medicine 2007 37 1 9 10.2190/41X8-42QW-2571-H20G 17645193

[B9] Loeppke R Taitel M Haufle V Parry T Kessler R Jinnett K Health and Productivity as a Business Strategy: A Multi-employer Study Journal of Occupational & Environmental Medicine 2009 51 411 428 10.1097/JOM.0b013e3181a39180 19339899

[B10] Nuttall M van der Meulen J Emberton M Charlson scores based on ICD-10 administrative data were valid in assessing comorbidity in patients undergoing urological cancer surgery J Clin Epidemiol 2006 59 265 273 10.1016/j.jclinepi.2005.07.015 16488357

[B11] Fortin M Bravo G Hudon C Lapointe L Dubois M-F Almirall J Psychological Distress and Multimorbidity in Primary Care Annals of Family Medicine 2006 5 417 422 10.1370/afm.528 PMC1578652 17003141

[B12] Fortin M Hudon C Dubois M-F Almirall J Lapointe L Soubhi H Comparative assessment of three different indices of multimorbidity for studies on health-related quality of life Health and Quality of Life Outcomes 2005 3 10.1186/1477-7525-3-74 PMC1310518 16305743

[B13] Hudon C Fortin M Vanasse A Cumulative Illness Rating Scale was a relaible and valid index in a family practice context Journal of Clinical Epidemiology 2005 58 603 608 10.1016/j.jclinepi.2004.10.017 15878474

[B14] Kessler R Barber C Beck A Berglund P Cleary P McKenas D Pronk N Simon G Stang P Ustun T Wang P The World Health Organisation Health and Work Performance Questionnaire (HPQ) Journal of Occupational and Environmental Medicine 2003 45 156 174 10.1097/01.jom.0000052967.43131.51 12625231

[B15] Kessler R Andrews G Colpe L Hirip E Mroczek D Normand S Walters E AM Z Short screening scales to monitor population prevalences and trends in non-specific psychological distress Psychological Medicine 2002 32 959 976 10.1017/S0033291702006074 12214795

[B16] Kessler R Barker P Colpe L Epstien J Gfroerer J Hiripi E Howes M Normand S-L Maunderscheid R Walters E Zaslavsky A Screening for Serious Mental Illness in the General Population Archives of General Psychiatry 2003 60 184 189 10.1001/archpsyc.60.2.184 12578436

[B17] Muthen L Muthen B MPlus: Statistical analysis with latent variables: Users Guide 2007 5 Los angeles: Muthen & Muthen

[B18] Tabchnick B Fidell L Using Multivariate Statistics 2006 5 Boston: Allyn & Bacon

[B19] Byrne B Structural equation modeling with AMOS: Basic concepts, applications and programming 2009 2 New York: Routledge

[B20] Appendix 1 & 2; Patterns and prevalence of multimorbidity in Australia http://www.fmrc.org.au/publications/appendices/

[B21] Cornell JE Pugh JA Williams JW Kazis L Lee AFS Parchman ML Zeber J Pederson T Montgomery KA Hitchcock Nokl P Multimorbidity clusters: Clustering binary data from multimorbidity clusters: Clustering binary data from a large administrative medical database Applied Multivariate Research 2007 12 163 182

[B22] Hudon C Fortin M Soubhi H Abbreviated guidelines for scoring the Cumulative Illness Rating Scale (CIRS) in family practice Journal of Clinical Epidemiology 2007 60 Letter to the Editor 10.1016/j.jclinepi.2005.12.021 17208130

[B23] Sundararajan V Henderson T Perry C Muggivan A Quan H Ghali WA New ICD-10 version of the Charlson comorbidity index predicted in-hospital mortality J Clin Epidemiol 2004 57 1288 1294 10.1016/j.jclinepi.2004.03.012 15617955

[B24] Rius C Perez G Martinez JM Bares M Schiaffino A Gispert R Fernandez E An adaptation of Charlson comorbidity index predicted subsequent mortality in a health survey J Clin Epidemiol 2004 57 403 408 10.1016/j.jclinepi.2003.09.016 15135843

[B25] Harse JD Holman CD Charlson's Index was a poor predictor of quality of life outcomes in a study of patients following joint replacement surgery J Clin Epidemiol 2005 58 1142 1149 10.1016/j.jclinepi.2005.02.017 16223657

[B26] Groll DL To T Bombardier C Wright JG The development of a comorbidity index with physical function as the outcome J Clin Epidemiol 2005 58 595 602 10.1016/j.jclinepi.2004.10.018 15878473

